# Stem Cells and Their Derivatives in Cardiac Fibrosis Therapy: Challenges and Perspectives

**DOI:** 10.3390/cells15080656

**Published:** 2026-04-08

**Authors:** Adrian Piwowar, Zuzanna Zolbach, Julia Rydzek, Natalia Skonieczna, Katarzyna Rojek, Mateusz Żołyniak, Julia Soczyńska, Sławomir Woźniak

**Affiliations:** 1 Student Scientific Group of Heart Diseases, Wroclaw Medical University, 50-556 Wroclaw, Poland; zuzanna.zolbach@student.umw.edu.pl (Z.Z.); julia.rydzek@student.umw.edu.pl (J.R.); natalia.skonieczna@student.umw.edu.pl (N.S.); katarzyna.rojek@student.umw.edu.pl (K.R.); mateusz.zolyniak@student.umw.edu.pl (M.Ż.); julia.niznik@student.umw.edu.pl (J.S.); 2 Student Scientific Society Anatomia-Klinika Nauka, Division of Anatomy, Department of Human Morphology and Embryology, Wroclaw Medical University, 50-367 Wroclaw, Poland; 3 Division of Anatomy, Department of Human Morphology and Embryology, Wroclaw Medical University, 50-367 Wroclaw, Poland; slawomir.wozniak@umw.edu.pl

**Keywords:** stem cell, cardiac fibrosis, stem cell derivatives, exosome

## Abstract

**Highlights:**

**What are the main findings?**

- Cell-based therapies, which may be potentially useful in the treatment of structural cardiac damage, are limited by challenges such as low cell survival rates and the risk of immune rejection.
- The use of exosomes may emerge as a preferred therapeutic strategy due to their low immunogenicity and lack of tumorigenic potential.

**What are the implications of the main findings?**

- The implementation of clinical trials with a long-term perspective is of critical importance.
- Particular attention should be paid to the personalization of therapy, including, among other factors, the selection of cell types and administration strategies.

**Abstract:**

Cardiac fibrosis is a pathology induced by various conditions, such as myocardial infarction, or certain cardiomyopathies, and represents one of the most prevalent cardiac abnormalities. This process, defined as the excessive accumulation of extracellular matrix within damaged cardiac tissue, leads to significant complications, including impaired systolic and diastolic function as well as arrhythmias. Conventional therapies focus primarily on slowing down the progression of fibrosis. Recently, there has been a growing research interest in therapies based on stem cells and their derivatives, which hold the potential to greater decrease formation and area of fibrosis. In this review, we aim to systematise the most recent data regarding the application of these approaches. We focus on describing the types of cells employed, methods of their implementation, and strategies for optimising these processes. Particular attention is given to exosomes due to the reports highlighting their use as innovative and potentially effective tools in the treatment of cardiac diseases.

## 1. Introduction

Cardiac fibrosis is a common cardiac muscle pathology caused by numerous heart diseases such as myocardial infarction (MI), aortic stenosis, hypertrophic cardiomyopathy and dilated cardiomyopathy [1,2]. According to Almehmadi et al. patterns of cardiac fibrosis are present in as many as 78% of patients with systolic dysfunction [3]. Moreover, Yeo et al. reported that 32.7% of patients with cardiometabolic conditions such as hypertension, obesity, hyperlipidaemia and diabetes suffer from cardiac fibrosis [4]. Considering that, cardiac fibrosis emerges as one of the most prevalent cardiovascular pathologies.

Cardiac fibrosis is defined as a process of excessive accumulation of extracellular matrix (ECM) within the tissue of damaged heart [5,6]. These deposits form scar-like structures, which are less elastic and less functional than healthy tissue. The main components of ECM deposited inside myocardium are type I and type III collagen fibres, which are secreted mainly by myofibroblasts [7,8]. Based on the location of ECM deposits, cardiac fibrosis can be divided into two major types: reactive fibrosis and replacement fibrosis. In reactive fibrosis, diffuse strands of collagen fibres are accumulated in interstitial and perivascular areas, and they form as a result of long-term mechanical strain on the heart tissue. In contrast, replacement fibrosis is characterised by the presence of focal scars composed of collagen fibres and caused by both ischaemic and non-ischaemic injuries of cardiac muscle [5,8,9].

Cardiac fibrosis leads to several significant complications. Excessive deposition of ECM stiffens the myocardium, which in turn impairs both systolic and diastolic motion of the heart. Furthermore, cardiac fibrosis is associated with ventricular arrhythmias as scar tissue disrupts cardiac electrical activity and electrophysiological harmony of myocardium. This is due to the fact that ECM propagates electrical impulses slower than healthy myocardium [10,11,12]. Because of these severe complications cardiac fibrosis has a crucial influence on the development of heart failure.

Current therapies of cardiac fibrosis focus on slowing down the progression of tissue fibrosis rather than eliminating existing ECM deposits. Drugs most commonly used for this purpose are: angiotensin-converting enzyme inhibitors, angiotensin receptor blockers, mineralocorticoid receptor antagonists, β-blockers and statins [1,8,13]. At the same time, new types of therapies, such as epigenetic therapies and biotechnological approaches, are emerging. They are focusing on decreasing formation and reducing total area of pathologic process in a damaged heart. Out of these novel treatment options, most notably stem cell-based therapies and therapies based on stem-cell secreted-factors are explored [1,14,15].

We aim to systematise the most recent data available in the literature regarding stem cells and their derivatives in the therapy of cardiac fibrosis. Our focus is on describing the types of cells used and the methods of their implantation. We highlight opportunities for optimising therapies utilising these cells. Regarding their derivatives, we primarily describe exosomes, given the reports of their use as innovative and potentially effective tools in the treatment of heart diseases. We identify the potential advantages and unfavourable aspects of such therapies. Furthermore, we pinpoint existing knowledge gaps, and outline potential directions for future research on stem cells and exosomes in the treatment of cardiac fibrosis.

## 2. Materials and Methods

Articles relevant for presented review were identified by conducting search in PubMed (https://pubmed.ncbi.nlm.nih.gov/), Scopus (https://www.scopus.com/) and Google Scholar (https://scholar.google.com/) data-bases. We limited our studies to articles published in English, between January 2014 and December 2025. Manual search with key words, such as “cardiac fibrosis”, “stem cells”, “exosomes”, “secretome”, “miRNA” or combinations of the above, were used to collect applicable papers. We screened abstracts of those to select articles eligible to this research. In the selected studies, cardiac fibrosis was caused mainly by MI but also by viral myocarditis, dilated cardiomyopathy, chronic heart failure, diabetic cardiomyopathy, pressure overload, or drugs, e.g., isoproterenol [16,17,18,19,20,21,22,23].

## 3. Stem Cell-Based Therapy

### 3.1. Mechanisms of Action and Delivery Techniques

Stem cells are cells capable of self-renewal and multipotent differentiation, found mostly in both connective and interstitial tissue [20,23]. In cardiac fibrosis therapy, researchers are using mostly mesenchymal stem cells (MSCs) such as bone marrow stem cells (BMSCs), adipose-derived stem cells (ADSCs), human amniotic MSCs or human umbilical cord MSCs (hucMSCs) [24,25]. There are also works exploring the use of cardiac progenitor cells (CPCs), muscle-derived stem cells, urine-derived stem cells, human amniotic fluid-derived mesenchymal stem cells (hAFMSCs) or human amniotic epithelial stem cells [26,27,28,29].

The use of stem cells is based on the process of remuscularization. Stem cells capable of differentiating into cardiac and vascular lineages, as well as reprogrammed fibroblasts, are employed. Authors highlight the direct implantation of cells as a dynamically evolving area of regenerative medicine [30]. Mechanisms associated with cardiac cells therapy encompass not only the survival of implanted cells, but also the stimulation of angiogenesis and vasculogenesis [31].

There are a few possible ways of administrating stem cells. Intravenous injections are often used due to low invasiveness. However, this method results in poor engraftment of the cells, which is exacerbated by specific mechanical characteristics of a heart [20,32,33,34,35]. In that case, other routes of administration were proposed, including transendocardial, intramyocardial and intracoronary injections [27,36,37]. Moreover, some researchers create stem cell tissue sheets, that are later turned into patches and adhered to the heart. Those sheets are constructed on a scaffolding made of biomaterials such as porous antioxidant polyurethane, poly(lactic-co-glycolic acid) fibre, poly(ethylene glycol) dimethacrylate solution, or electrospinning cellulose nanofibers modified with chitosan/silk fibroin. Those routes’ main advantage is that cells are delivered closer to the damaged area of the heart [28,32,34,38].

Despite the aforementioned advantages of more invasive techniques, Mokhtari et al. states that intravenous injections improve heart function at the same level as intramyocardial ones [39].

Another described way of stem cells administration is pericardial application. Zhang et al. proposed this route for stem cell cardiospheres, which cannot be injected in other ways due to possible embolism [40].

Some authors propose that repeated intravenous injections can be more beneficial than administrating only one dose. They managed to achieve significant increase in ejection fraction (EF) and decrease in collagen content in damaged heart. A study by Tang et al. showed that three infusions of MSCs led to an increase in EF of approximately 7.9%, compared to the pre-treatment state. In a single-dose trial, only a small increase in EF was observed, indicating the higher efficacy of repeated intravenous injections [41].

### 3.2. Strategies for Optimising Therapeutic Approaches

As stem cell-based therapy is still a novel type of treatment, most of presented research was conducted on animal models, notably rats and mice, unless stated otherwise.

One of the main problems of a stem cell therapy are poor cell survival rates. It is attributed to a hostile environment in the heart, caused by ischaemia, hypoxia and oxidative stress. Some authors see it as a result of inflammation or, when it comes to allogenic cells, immunological rejection [42,43,44]. To fight this obstacle, various measures are undertaken. One of them is to achieve anti-inflammatory effect by using specific drugs—inhibitors of soluble epoxide hydrolase enzyme [43]. Another measure is using biomaterial scaffolds as vehicles during injections. Chen et al. used transglutaminase cross-linked gelatin in intramyocardial injection of ADSCs and managed to increase both retention and cardioprotective functions of these cells [45]. Other way of enhancing cell survival is by preconditioning them with hydrogen sulfide. Abdelmonem et al. used these kind of cells and achieved better survival rates as well as increased homing, better improvement of heart function and reduction in TGF-β1 levels [46].

Alternative method includes modifying genome of transplanted stem cells. Cai et al. managed to achieve *HAX1* genes overexpression in CPCs, which resulted in enhanced cell survival. In turn, Cho et al. introduced *LEF1* gene into hucMSCs, managing to increase proliferation and cell survival [47,48]. Han et al. transfected BMSCs with *YTHDC1* gene, achieving its overexpression and apoptosis reduction [49]. Other researchers managed to suppress pyroptosis, which was identified as one of main processes causing stem cell deaths after transplantation, by transfecting cells with miRNA-762. This action increased survival rate of the cells [42].

When it comes to immunological rejection, Shao et al. tried to overcome it by disrupting the gene encoding β-2 microglobulin. This protein is part of HLA-I, which is responsible for CD8+ T lymphocytes attacking transplanted cells. Knockout of β-2 microglobulin gene successfully prevented immunological rejection [44]. It should be noted that hAFMSCs express low amount of MHC-I antigens and none MHC-II antigens and, therefore, are low-immunogenic [50].

Methods of preconditioning, resulting in increased survival rate of stem cells are presented in Table 1.

Gene modification can also have an impact on overall outcome of stem cell therapy. MSCs were modified by adding adiponectin or adrenomedullin genes via transduction. Both research managed to decrease cardiac fibrosis and, therefore, improve heart function [52,53]. Moreover, Cao et al. transfected BMSCs with miRNA-133a and achieved inhibition of cardiac fibrosis and even improvement in transplanted cells’ survival [51].

Researchers also develop other strategies in order to increase therapeutic effects of stem cell therapy. Xuan et al. managed to induce differentiation of CPCs into several cardiac lineage cells with use of isoxazole. This approach, which takes advantage of stem cells’ ability to develop into other cells was successful: LVEF was increased to 71.95 ± 1.53% from 43.39 ± 2.31% in a group treated with CPCs [26]. Anti-inflammatory macrophages also play a crucial role in the process of heart healing. Some researchers tried to include them in stem cells therapy, either by co-culturing them with used cells or by preconditioning cells with n-butylidenephthalide, which activates M2 macrophages. Both research managed to achieve heart function improvement [54,55].

Another way of improving therapy outcome is to precondition stem cells with substances such as irisin, which is newly identified cardiokine, or MHY-1685—small molecule that can inhibit mTOR signalling. Both compounds manage to reduce interstitial fibrosis and improve cardiac repair, while the latter also exhibits potential to differentiate into endothelial and smooth muscle cells (this effect is observed on in vitro model) [56,57]. Moreover, Chen et al. pretreated human amniotic MSCs with S100a8 and S100a9, which are calcium-binding proteins. This action resulted in decreasing of myocardial fibrosis and improvement of heart function [25].

There is some research suggesting that medications currently used in therapy of cardiac fibrosis, β-blockers and angiotensin receptor blockers, may interfere with stem cell-based therapies. Carvedilol and irbesartan were studied. Both drugs were administered one week before and five weeks after inducing MI. Each study showed that treatment with these medications resulted in lower EF and higher percentage of fibrosis area compared to the group treated with ADSCs sheets alone [58,59].

## 4. Therapies Based on Stem Cell-Secreted Factors

### 4.1. Exosomes as Key Mediators

According to preclinical evidence, the role of the cellular secretome may potentially contribute to cardiac muscle regeneration. Therefore, a distinction should be made between soluble and insoluble factors [30,60]. Soluble protein fraction includes, among others, growth factors and cytokines [61]. In the context of the heart and vasculature, factors such as VEGF, IGF-1, hypoxia-inducible factor 1, as well as IL-1 and IL-6, are most relevant. Their mechanisms include the promotion of neovascularization, inhibition of fibrosis, and the stimulation of stem cell differentiation [62].

When it comes to insoluble factors, extracellular vesicles (EVs) such as exosomes, apoptotic bodies, and microvesicles have been identified [63,64].

The literature reports that, due to existing limitations such as separation technologies, exosomes remain the most extensively studied type of EVs [64]. For this reason, they have been the primary focus of our attention.

Exosomes are described as membrane-bound nanoparticles [65]. Characteristically, they contain a wide range of biomolecules, including proteins, lipids, DNAs, mRNAs and/or miRNAs. Their primary function is to serve as effective components of cell-to-cell communication [66,67]. The amount of exosomes produced by a cell depends on its type: each type secretes exosomes with different protein levels in cargo and/or in bilayer membranes [68]. Exosomes currently used in research are derived from multiple stem cell types including: MSCs, BMSCs, hAFMSCs, hucMSCs, ADSCs, human induced pluripotent stem cells (hiPSCs) and induced pluripotent stem cells (iPSCs).

One of the most abundant components of exosomes are miRNAs, which are small, stable, non-coding RNA molecules [69]. Studies have shown their role in cell-to-cell communication and regulating the expression of a variety of mRNAs [70]. Recent research revealed that changes in miRNA expression may lead to cardiovascular diseases. However, above that, many studies have shown therapeutic effects of miRNA molecules delivered by exosomes in MI injury [66,71]. Delivery of miRNA to cardiac cells is being intensively researched. Some of the promising ways of delivery include carriers based on adeno-associated virus or lipocomplexes. Unfortunately, these approaches have clinical limitations, such as high cytotoxicity, potential immunogenicity and instability in the blood and serum [72,73]. Usage of exosomes as vehicles is a really promising method of exogenous administration of miRNAs. As natural carriers, they have certain advantages such as low immunogenicity, high efficiency of intracellular transport, relatively long blood half-life, and low cytotoxicity [74,75].

In the following sections, the effects of exosomes derived from various cell types are presented. All of the presented research was conducted on animals, notably mice and rats, unless stated otherwise.

#### 4.1.1. hucMSCs-Derived Exosomes

Yang et al. explored miRNA-223 delivery by using hucMSCs-derived exosomes (hucMSCs-exos) in an MI model. They determined that P53 protein, which transcriptionally regulates S100A9 expression, is a direct target of miRNA-223. This miRNA may modulate the P53/S100A9 axis, thereby inhibiting cardiac fibrosis and promoting angiogenesis [71]. Additionally, hucMSCs-exos transporting miRNA-29b exhibited anti-fibrotic effects. These results were especially distinct in a group treated with exosomes loaded with miRNA-29b mimics [76]. Studies have also shown that intravenous administration of hucMSCs-exos following acute MI, can improve LVEF and left ventricular fractional shortening (LVFS), while also decreasing left ventricular inner diameter (LVID) and, in some models, left ventricular end-diastolic dimensions (LVEDD). Moreover, hucMSCs-exosome therapy reduces myocardial fibrosis and cardiomyocyte apoptosis, effects that are not seen with hucMSCs-conditioned medium depleted of exosomes [77,78]. Furthermore, Zhu et al. demonstrated that engineering macrophage migration inhibitory factor into hucMSCs can enhance the effects of exosomes secreted by these cells, presumably via upregulation of miRNA-133a-3p expression and activation of AKT signalling [79].

#### 4.1.2. ADSCs-Derived Exosomes

ADSCs-derived exosomes (ADSCs-exos) generate great research interest. In an in Vivo and in Vitro experiments, ADSCs-conditioned medium, containing ADSCs-exos and other paracrine factors, was applied to ischaemic hearts and hypoxic cardiomyocytes. This conditioned medium contained abundant miRNA 221/222, which are downregulated in infarcted areas. Treatment reduced cardiac fibrosis and apoptosis and increased miRNA-221/222 levels in ischaemic tissue, modulating PUMA/p53/BCL2 and ETS-1/fibronectin/collagen 3 pathways via p38/NFκB and thereby attenuating pathological remodelling. However, purified exosomes were not used, so the observed mechanisms may also involve non-exosomal components of the ADSCs medium [80]. Studies have also established that administration of ADSCs-exos enriched in miRNA-205 improves EF in MI models, while simultaneously reducing the area of cardiac fibrosis in comparison to control groups [67]. Moreover, ADSCs-exos obtained from young mice decrease the amount of age-related fibrotic tissue in hearts of old mice [81]. ADSCs overexpressing miRNA-126 secrete exosomes that alleviate myocardial injury by downregulating TNF-α, IL-1β and IL-6, and enhancing angiogenesis, thereby improving cardiac function [82]. Notably, in a hypoxia-injured cardiomyocyte model, pharmacological inhibition of exosome release from ADSCs largely abolished their preservative properties. That indicates exosomes play a primary role in stem cell-based therapy through paracrine mechanisms [80,82].

Researchers identified some signalling pathways, which are involved in ADSCs-exos cardioprotective properties [80,83]. Upregulation of the S1P/SK1/S1PR1 pathway is one of the proposed mechanisms, which allows ADSCs-exos to reduce both levels of TNF-α, IL-1β, IL-6 and IFN-γ, and limit MI-induced fibrosis and apoptosis. At the same time, this signalling pathway is also involved in promoting M2-macrophage polarisation, reversing MI-induced M1-macrophage differentiation [83].

#### 4.1.3. BMSCs-Derived Exosomes

Yet another thoroughly researched exosomes are BMSCs-derived exosomes (BMSCs-exos). Based on both in vivo and in vitro studies, these exosomes may mitigate cardiac hypertrophy and cardiac remodelling [21]. Furthermore, BMSCs-exos have been shown to downregulate expression of collagen I, α-SMA or TGF-β1, which are considered to be fibrosis-related markers [84,85]. Upregulation of Nfr2 signalling is one of the proposed mechanisms of BMSCs-exos action. BMSCs-exos overexpressing Nrf2 further alleviated atrial fibrosis in comparison to unmodified exosomes [84]. Additionally, these exosomes inhibit the histone methyltransferase EZH2, thereby relieving repression of HMGA2 and activating PI3K/AKT signalling, which contributes to their anti-fibrotic effect [86]. Another study used BMSCs-exos to deliver miRNA-19a/19b to cultured HL-1 cardiomyocytes, resulting in downregulation of pro-apoptotic genes (Bim and PTEN) in comparison to the control group [87].

#### 4.1.4. hiPSCs- and iPSCs-Derived Exosomes

When it comes to hiPSCs-derived exosomes (hiPSCs-exos) and iPSCs-derived exosomes (iPSCs-exos), studies have shown not only their anti-fibrotic and anti-inflammatory potential but also their excellent safety profile [88,89]. Adamiak et al. compared effects of intramyocardial injections of iPSCs and iPSCs-exos. Results included greater reduction in apoptosis and greater improvement in left ventricular function at 35 days after coronary occlusion and subsequent reperfusion in the group injected with iPSCs-exos. Additionally, iPSCs improved systolic wall thickness by 10%, while exosomes by 19% in comparison to control group. In the same study, iPSCs injection resulted in teratoma development in 53% cases, whereas iPSCs-exos injections did not cause a single neoplastic transformation [88]. Contents of hiPSCs-exos can be modified by altering the amount of oxygen in hiPSCs’ environment. Studies have established that hiPSCs-exos obtained from hypoxic conditions (5% O_2_), show elevated miRNA-302b-3p expression, which is associated with greater anti-fibrotic effect [89].

hiPSCMs also secrete exosomes. Some studies indicate that exosomes coming from hiPSCs-derived cells (e.g., hiPSCMs-derived exosomes (hiPSCMs-exos)) present more promising therapeutic effects, compared with exosomes isolated from tissue-derived stem cells. Tzng et al. observed that 4 weeks after the ischaemic injury, a group administered with hiPSCMs-exos had significantly improved myocardial viability (median: 92.48% [IQR, 87.72–95.46%]; *p* < 0.05) compared with the control group (median: control, 79.21% [IQR, 77.82–81.14%]). At the same time MSCs-derived exosome treatment did not significantly increase myocardial viability [90].

hiPSCMs-exos contain a variety of miRNAs, including molecules associated with antifibrosis, angiogenesis and M2 macrophage polarisation. Their exosomal molecular mechanism of action consist in upregulation of Hgf, Il4, Il10, Il13, expression of Tgfb1 and elevation of Pdgfb, Igf1, Hgf, and Sdf1 mRNA levels [91].

hiPSCs-derived MSCs are another type of hiPSCs derivatives, which secrete exosomes presenting anti-fibrotic and proangiogenic effects in ischaemic myocardium [92].

#### 4.1.5. hAFMSC-Derived Exosomes

Another type of exosomes—hAFMSC-derived exosomes—are reported to relieve the ECM deposition and increase the expression levels of HIF-1α and VEGF in fibrotic heart [22].

Effects of RNAs delivered through aforementioned types of exosomes are presented in Table 2.

### 4.2. Exosome Delivery Methods

In research settings exosomes are typically administered intravenously, e.g., via the tail vein in mouse model or intramyocardially [21,22,67,77,91].

It has been established that retention of exosomes within the heart was much greater after intramyocardial delivery compared with intracoronary injection. Therapeutic effects after intracoronary administration were minimal [75]. Intravenous injection has generally been less effective than intramyocardial delivery. Preclinical studies show little or no cardiac localization and minimal therapeutic benefit after intravenous administration, whereas intramyocardial injection results in robust myocardial exosome retention and clear functional improvement [98].

Intravenously administered exosomes are quickly cleared from blood circulation, with reported terminal half-lives in the range of roughly 70–180 min. These exosomes are mainly distributed to the liver, spleen and lungs, which markedly limits the amount reaching the heart [99,100]. Macrophages are primarily responsible for clearance of the exosomes [68].

Current strategies of increasing exosome delivery to the heart include engineering exosomal transmembrane proteins displaying targeting peptides [101,102]. The CSTSMLKAC peptide sequence is known to target ischaemic myocardium, which makes it a promising candidate for fusion with exosomes. As expected, pure exosomes bind to injured cardiomyocytes less effectively than exosomes fused with CSTSMLKAC peptide (38.66 ± 0.86% versus 43.96 ± 1.21%) [102]. Connexin 43 present on exosomal membranes has also been said to improve intercellular communication and may be utilised to further enhance exosome-mediated delivery [101]. Researchers also developed cardiac homing peptide attached to exosomes, which increases ischaemic myocardium targeting and reduces off-target binding. Cardiac-homing-peptide-tagged exosomes showed higher retention in fresh neonatal rat cardiomyocytes in comparison to control group [103].

Intramyocardial administration of exosomes can be achieved by direct injections or by injectable hydrogels encapsulating exosomes [104]. The first approach has low effectiveness due to the limited retention of exosomes in the ischaemic tissue [65]. Repeated intramyocardial injections could in principle overcome low cardiac exposure; however this strategy would be invasive and burdensome. Preclinical data suggest that different systemic delivery routes of exosomes can achieve comparable efficacy, allowing route selection to minimise patients’ burden [105]. Although intramyocardial injections are widely used in animal studies, in humans this approach typically requires catheter-based or surgical access to the myocardium, which is highly invasive and therefore less than ideal for clinical application [103].

On the other hand, injectable hydrogels encapsulating exosomes can improve exosome delivery to the infarct area and extend their retention time [104,106]. Hydrogels are three-dimensional, crosslinked hydrophilic polymer networks with high biocompatibility [107]. They have some promising features such as the ability to mimic natural ECM, easily controlled physical properties and remarkable ductility [106,108]. There are reports of greater myocardial function recovery after therapy based on hydrogel-encapsulated exosomes compared to treatment based on free exosomes [85,108,109]. Researchers have developed and tested numerous types of hydrogels, including injectable thermosensitive hydrogels, shear-thinning hydrogels, hydrogel patches and nanocomposite hydrogels [108,109,110,111]. Moreover, studies continue to incorporate peptides like angiogenin-1 or CP05 peptide into hydrogels to improve their effectiveness [104,108,112].

The most practical approach to hydrogel properties in heart disease is to prioritise their ability to release exosomes over a prolonged period of time. Studies show that hydrogels are capable of sustained in vivo delivery of exosomes up to 21 days in some systems [110,113].

There are also studies demonstrating that hydrogels as well as exosomes can be delivered via the intrapericardial route in rodent models [18,112,114]. This approach is especially relevant in cardiac fibrosis due to the fact that cardiac fibroblasts originate from epicardium [112].

Inhalation delivery of exosomes is the newest proposed approach. According to Li et al., seven consecutive days of nebulisation therapy can result in reduced cardiac fibrosis area and improved left ventricle function in MI models. Mouse models showed a reduction in size of ischaemic area and an increase in EF of approximately 11.7% (Δ = 11.66 ± 5.12%) compared to control groups [115].

**Figure 1:**
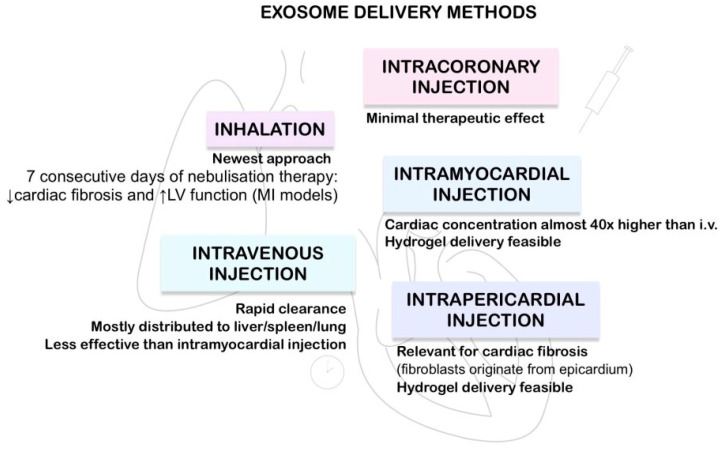
Exosome Delivery methods [68,75,98,104,112,114,115].

The summary of exosome delivery methods is presented in Figure 1.

## 5. Discussion

### 5.1. Standardisation of Stem Cell- and EV-Based Therapies

Unlike conventional therapies, the preparation of stem cells and EVs requires advanced laboratory technologies, specialised infrastructure and qualified personnel, which significantly increases the cost of the entire process [116,117,118,119]. Clinical-scale cell production requires appropriate culture conditions, quality control and biosafety, as well as compliance with stringent regulatory requirements [118,119,120].

Standardisation represents one of the key prerequisites for the successful clinical translation of stem cell-based and EV-based therapies [121]. In contrast to conventional pharmacological agents, biological products such as stem cells and their derivatives are characterised by considerable variability related to donor characteristics, cell source, culture conditions, and manufacturing protocols [122]. This variability complicates both the reproducibility of experimental findings and the comparison of results across studies [123].

From a regulatory perspective, agencies such as the European Medicines Agency and the U.S. Food and Drug Administration, classify most stem cell-based therapies as advanced therapy medicinal products, which requires strict adherence to good manufacturing practice standards, validated production protocols and well-defined quality control parameters [124]. These parameters typically include cell identity, purity, viability, genetic stability, and functional potency [125]. To ensure aforementioned parameters are fulfilled, cells undergo rigorous testing. Flow cytometry confirms presence of specific markers. Whole process is conducted in aseptic environment to ensure minimal level of endotoxins [126]. To ensure storage stability, cells undergo cryopreservation, during which they are covered in DMSO-free cryoprotectants, ensuring minimal toxicity and improving post-thaw recovery [127,128].

For EVs, the challenge of standardisation is even more pronounced due to the heterogeneity of vesicle populations and the diversity of isolation techniques [129]. International scientific societies have, therefore, proposed minimal reporting frameworks such as the Minimal Information for Studies of EVs guidelines [130]. These recommendations emphasise the need for consistent reporting of EV isolation methods, particle sizing and counting, and the presence of characteristic molecular markers [131].

The adoption of standardised reporting criteria is essential for improving reproducibility, enabling meaningful comparison between studies and facilitating regulatory approval of future regenerative therapies [132].

Important aspect of standardisation concerns the implementation of functional potency assays. For both stem cell-based and EV-based therapies, potency assays should be closely aligned with the proposed mechanism of therapeutic action [133]. In the context of cardiac fibrosis, appropriate assays may include evaluation of cardiac fibroblast activation and proliferation, inhibition of myofibroblast differentiation (e.g., α-SMA expression), reduction in type I and III collagen synthesis, or modulation of pro-fibrotic signalling pathways like TGF β/Smad pathway [134,135,136].

Such functional assays provide direct evidence that the biological product exerts the intended anti-fibrotic activity and are increasingly required by regulatory authorities as part of quality control strategies for advanced biological therapies [137,138].

Although exosome-based therapies may overcome several limitations associated with direct cell transplantation, important translational challenges remain.

To facilitate comparison between studies and improve reproducibility, key methodological parameters that should be reported in stem cell and EVs research are summarised in Table 3. These minimum reporting items include information on cell source, culture conditions, potency assays, EV isolation and characterisation methods, as well as dosing definitions and administration protocols.

Need for standardisation also applies to conducted research. When it comes to comparison of aforementioned studies as well as drawing conclusions from them, it is important to acknowledge the distinction in methodology between them. Main differences are: causes of cardiac fibrosis and methods of its quantification and methods of isolating exosomes, their characteristics and doses.

Main cause of fibrosis in presented results is MI, where excessive collagen accumulation in ECM is a result of scarring due to ischaemia. However, some studies rely on different fibrosis triggers, such as pressure overload, diabetic cardiomyopathy or artificially induced fibrosis (e.g., by isoproterenol), where mechanisms leading to accumulation of ECM are different [17,20,21,22]. As it may impact the response to the therapy, it should be taken into consideration while comparing the results.

There are also different ways of assessing the extent of fibrosis process. Quantification is done based on methods such as Masson’s trichrome staining or RT-qPCR, Western blot and immunofluorescence staining for fibrosis markers like type I and III of collagen, α-SMA or fibronectin mRNA/protein [67,76,79]. Those methods differ in sensitivity and specificity as well as in objectivity, with staining being more subjective as it relies strongly on abilities of researchers and on area of the examined tissue. Those distinctions should be taken into account during the comparison of the results.

It should also be noted that every aforementioned study concerning exosomes has its own standards when it comes to isolation, characteristics and dosage, as there are no generally accepted standards yet. For this reason, comparison among them should be made cautiously.

### 5.2. Challenges of Stem Cell Therapy

Stem cells therapy for the treatment of cardiac fibrosis, although promising, is associated with significant risks that distinguish it from conventional treatments [153,154]. One of the most serious problems is the risk of tumour formation, especially when using pluripotent cells such as iPSCs [155,156]. Due to their ability to divide and differentiate indefinitely, these cells can lead to the formation of neoplasms if immature cells remain in the preparation. Studies have shown that even a small number of undifferentiated iPSCs transplanted into the heart can result in tumour development, and this risk depends not only on the number of cells administered, but also on their quality and degree of differentiation [157,158].

Another potential threat is the risk of immune reactions. In the case of allogeneic transplants, i.e., those from another donor, there is a possibility of cell rejection by the recipient’s immune system, which can lead to inflammation, transplant damage or the need for immunosuppression, which in turn increases susceptibility to infections [116,154,156]. Even in the case of autologous cells, i.e., those derived from the patient, the process of reprogramming and differentiation can lead to the formation of new antigens that trigger an immune response [156].

In addition, stem cells therapy is associated with a risk of thrombotic complications. The administration of cells, especially intravenously, can lead to the activation of the coagulation system and the formation of blood clots, which pose a serious risk to the patient. In the case of MSCs, this risk is lower than with iPSCs, but it is not completely eliminated [159,160].

After transplantation, cells such as MSC- or iPSC-derived cardiomyocytes encounter the hostile microenvironment of the damaged heart, characterised by hypoxia, oxidative stress, inflammation and a lack of adequate ECM [159,161]. These factors lead to rapid cell death and ‘washout’ from the site of administration, leaving less than 10% of cells in the heart tissue after 24 h, with only 1% surviving in the long term [34,162,163,164]. However, it is important to note that, currently, scientists conduct successful research in the department of preconditioning to overcome this problem.

Furthermore, the differentiation of stem cells into mature and functional cardiomyocytes is observed to be inefficient [165,166]. Current differentiation protocols often lead to the formation of immature cells with heterogeneous properties that do not match the functionality of native heart cells [167,168]. In addition, transplanted cells show poor integration with the host tissue, meaning that they do not form effective electrical and mechanical connections with the surrounding heart muscle [162,169]. Such incomplete integration promotes the development of conduction disturbances and can lead to arrhythmias, including life-threatening ventricular tachyarrhythmias [166,170]. The risk of arrhythmia is particularly high when the transplanted cells are immature or exhibit pacemaker cell characteristics, and when integration with the host is incomplete or irregular [171,172,173].

The results of clinical trials of stem cells therapy vary widely, with some showing improvement in heart function and others failing to confirm significant benefits [153,162]. These differences are due to different cell sources, preparation methods, timing of administration, and individual patient characteristics [174,175]. There is no clear evidence of the long-term effectiveness of this therapy as studies to date often involve short observation periods and small patient groups [176,177].

There are no standardised guidelines for the selection of cell type, dose, route of administration or duration of treatment [178,179]. Studies use different cell populations, isolation and administration methods, which makes it difficult to compare results and draw clear conclusions. For example, many iPSC-derived cardiomyocytes vary between themselves on functional level and also act differently than natural adult cardiomyocytes [180]. Moreover, comparison of CPC-, MSC-, and iPSC-derived cells highlight source-dependent efficacy in repair mechanisms like immunomodulation [181]. Standardisation of these elements is crucial to improve the reproducibility and effectiveness of the therapy [175,176].

### 5.3. Challenges of EV-Based Therapy

The treatment of cardiac fibrosis using exosomes has significant limitations due to their biological ambivalence. Endogenous exosomes can exacerbate fibroblast activation, cardiomyocyte hypertrophy, and adverse cardiac remodelling, while inhibition of their biogenesis (e.g., with tipifarnib) reduces fibrosis and improves cardiac function, indicating their potentially pathogenic role [182,183,184]. The molecular composition of exosomes, including miRNAs and proteins, is highly dependent on the type of stem cell and the stimuli acting on it. As a result, there is a significant risk of undesirable pro-inflammatory or pro-fibrotic biological cargo. In addition, numerous miRNAs exhibit context-dependent activity, which significantly limits the ability to predict their functional effects in the course of complex heart diseases [184,185,186]. Another major drawback is heterogeneity and standardisation: isolates contain a mixture of different EVs, their composition varies between batches and centres, and the lack of uniform, clinically accepted protocols for isolation, characterisation and potency testing, hinders the registration of biological drugs [187,188]. Pharmacokinetics is also problematic—after intravenous administration, a significant proportion of exosomes is captured by both liver and spleen, resulting in low delivery to the fibrotic myocardium and a risk of off-target adverse effects [17,188]. Clinical-scale production remains a major challenge: high yield, reproducibility, absence of contaminants and storage stability must be ensured, which is difficult and costly with naturally variable donor cells [65,187]. In addition, virtually all evidence of anti-fibrotic activity comes from rodent models after infarction, or from other animals, and data from controlled clinical trials of exosomes strictly against cardiac fibrosis is currently lacking. The first human trials mainly concern cells or their exosomes in heart failure in general, with small sample sizes and an emphasis on safety [65,189,190].

The summary of most common problems with stem cells and exosome-based therapies is presented in Table 4.

### 5.4. Comparison of Therapies Based on EVs and EV Mimetics

Due to the low yield and other challenges associated with the application of EVs, EV mimetic nanovesicles have been developed for clinical use [191]. The literature highlights the similarities between EVs and mimetics in terms of size, shape, RNA and protein composition, and low immunogenicity [192]. Compared to EVs, mimetics are characterised by higher yield, easier production, and improved uptake [191].

Studies by Huang et al. on EV mimetics have demonstrated their role in protecting cardiomyocytes from apoptosis. In murine models, EV mimetics achieve comparable results to natural EVs. It is anticipated that the miRNA-24-3p contained in EV mimetics contributes to enhanced antioxidant activity and inhibition of inflammatory responses in injured cardiomyocytes [193].

One study showed that Iron Oxide Nanoparticle–derived Nanovesicles, injected into the heart post-MI and directed magnetically, promoted an early transition to the regenerative phase, reduced apoptosis and fibrosis, and enhanced angiogenesis [194].

Further research is required to evaluate safety and to elucidate key regulatory pathways for the standardisation and quality control of production. Such efforts are essential to achieve high-quality, safe therapies based on EV mimetics [192]. In Table 5. we compare the limitations, advantages, and requirements of biological exosomes with their mimetics.

### 5.5. Potential Advantages of Stem Cell and Their Derivatives Therapy

Despite the aforementioned problems with both stem-cells and exosome-based therapies, there are many possible advantages of these treatments in comparison to classical approaches of cardiac fibrosis therapy. It stems from the unprecedented ability of preventing and reversing pathological remodelling of the heart, leading to cardioprotection [177].

The study conducted on rats with doxorubicin-induced dilated cardiomyopathy, using hUCMSCs injected intramuscularly, resulted in higher LVEF as well as the increase in VEGF, IGF-1 and HGF. Moreover, the amount of BNP decreased showing that hucMSCs can support cardiac function and help with the preservation of myocardium [199]. Another study, conducted in 2020 on pigs showed that intravenous dosage of MSCs lowered microvascular obstruction, helped to boost the LVEF. Furthermore, in histological overtake it showed smaller area of fibrosis and less inflammatory cells with the potential of reducing compromised infarct area [200]. Another research shows us the possibility of converting human stem cells into CPCs using isoxazole, which was proven successful to moderate fibrosis and enhance vascularity in mice [26].

It is important to bring out the fact that therapies based on stem cells and their derivatives are considered potentially effective in curing heart diseases mostly because of their paracrine effects, decreasing inflammation which results in generally preferable remodelling, encouraging repairs and protection of cardiac muscle. Particles believed to be responsible for that are cytokines and growth factors. EVs, particularly exosomes, play a significant role in mediating the paracrine effects [201].

Exosome-based therapies may offer several important advantages compared with direct stem cell transplantation [202,203,204]. Due to their nanoscale size, exosomes can penetrate tissues more efficiently and facilitate intercellular communication through the transfer of bioactive molecules such as proteins, lipids and regulatory RNAs [203,205,206]. Moreover, exosomes are associated with lower immunogenicity and improved safety profiles compared with living cell transplantation [202,205,207,208]. Importantly, numerous preclinical studies have demonstrated that administration of stem cell-derived exosomes can reproduce many of the beneficial paracrine effects of stem cells, including attenuation of cardiac fibrosis, reduction in inflammation, and promotion of angiogenesis [208,209,210]. It is also crucial to note that exosomes lack the capacity for self-replication; therefore, therapies based on their administration do not carry the risk of teratoma formation [189].

While implantation of stem cells still comes with many obstacles, proper preparation of those cells can increase their medical value, for ex., intracoronary MSCs with integrin-linked kinase were proven to increase both LVEF and angiogenesis, while slowing apoptosis and remodelling of heart tissue. This results in smaller scarring, fibrosis, and generally better performance of the cardiac muscle in comparison to other MSC types [211,212].

### 5.6. Development Opportunities and Future

Current research on stem cells and their derivatives in the treatment of cardiac fibrosis focuses on several innovative directions that aim to increase the efficacy, safety and personalisation of therapy [66,213]. Currently exosome-based therapies may become the preferred, safer alternative to classic stem cell transplants in the future [189,214]. This is the result of exosomes exhibiting potent anti-inflammatory, anti-apoptotic, pro-angiogenic and anti-fibrotic effects, while minimising the risk of immune and neoplastic reactions that may accompany direct cell transplantation [215,216].

Another important area of development is tissue engineering and the use of biomaterials that improve cell survival, retention and integration in the damaged heart [120,163]. Advanced biomaterials, such as 3D matrices, hydrogels and nanomaterials, enable the creation of a microenvironment conducive to regeneration, protect cells from oxidative stress and hypoxia, and support their functional integration with heart tissue. This approach allows for more effective delivery of cells and growth factors to the site of damage, increasing the chances of restoring heart muscle function [217,218,219].

There are also high hopes for genetic modifications and preconditioning of stem cells to increase their survival, differentiation capacity and resistance to the adverse conditions prevailing in the damaged heart [120,220]. Examples include transducing cells with genes encoding growth factors (e.g., VEGFA, bFGF), preconditioning with hypoxia or using pharmacological agents, which leads to a stronger therapeutic effect, better vascular regeneration and reduction in fibrosis [221,222,223].

Finally, the future of stem cell therapy lies in increasing personalization—selecting the type of cells, administration strategy and modifications to suit the individual characteristics of the patient, such as inflammatory profile, degree of heart damage or presence of comorbidities [120]. The use of artificial intelligence tools, multi-omic analysis and biobanks makes it possible to identify patients who respond best to specific therapies and to predict the efficacy and safety of treatment. Personalisation of therapy allows for maximisation of effects while minimising the risk of complications [175,224].

## 6. Conclusions

Therapies utilising stem cells and their derivatives may potentially play a role in the treatment of structural cardiac damage. Cell-based therapies; however, are limited by factors such as low cell survival rates and the risk of immune rejection. Consequently, exosome-based approaches may emerge as a preferred strategy in the future due to their low immunogenicity and absence of tumorigenic potential. The possibility of combining cell-based therapies with paracrine mechanisms should also be considered.

Future progress in this field will depend on the development of harmonised manufacturing protocols, standardised characterisation methods and clearly defined potency assays that ensure reproducibility and facilitate regulatory approval of therapies based on stem cells and their derivatives.

Promising preclinical results should be regarded as an initial prelude rather than definitive evidence. Although exosomes demonstrate promising therapeutic potential and may overcome several limitations associated with stem cell transplantation, further work is required to address challenges related to standardisation, large-scale production and regulatory approval. Attention must be directed toward the standardisation of procedures to ensure reproducibility and safety. The implementation of large-scale clinical trials, with an emphasis on long-term evaluation of therapeutic efficacy, is essential. Personalization also appears critical, encompassing the selection of cell types, administration strategies, and adaptation to individual patient profiles.

## Figures and Tables

**Table 1:** Methods of stem cells preconditioning.

Aim of Procedure	Type of Cells	Mechanism of Action	Results	Therapeutic Effects	References
**suppressing pyroptosis**	human ADSCs	delivery of exogenous miRNA-762 downregulates IL-1b expression and subsequently mitigates pyroptosis of stem cells	↑ survival rate of the cells	↑ EF ↓ area of fibrosis in comparison to the control group	[42]
**reduction in inflammation by administering soluble epoxide hydrolase inhibitor**	human-induced pluripotent stem cell-derived cardiomyocytes (hiPSCMs)	diminishes the pathological MAPK signalling cascade, reactive oxygen species, and apoptosis in administered cells, reducing oxidative stress and apoptosis of cardiomyocytes	↑ survival rate of the cells	↑ fractional shortening (FS) in comparison to the control group	[43]
**knock-out of β-2-microglobulin gene**	allogeneic hucMSCs	knock-out of β-2-microglobulin gene results in limiting function of HLA-I, which presents antigens to CD8+ T cells that can attack transplanted cells	prevention of immune rejection of the cells	↑ EF in comparison to the control group	[44]
**cellular delivery using transglutaminase cross-linked gelatine as scaffolding**	ADSCs	transglutaminase cross-linked gelatine provides microenvironment for maintaining survival and proliferation of cells in ischaemic heart tissue	↑ longevity of the cells survival	↑ EF ↓ area of fibrosis in comparison to the control group	[45]
**overexpression of *LEF1* gene**	human umbilical cord blood-derived MSCs	LEF1 expression protects cells from oxidative stress by increasing Bcl-2 expression	↑ longevity of the cell survival	↑ EF and FS ↓ area of fibrosis in comparison to the control group	[47]
**overexpression of *HAX1* gene**	cardiac stem cells	HAX1 inhibits activity of Mst1 kinase and modulates the Hippo–Yap pathway, which results in increased cell survival under hypoxic conditions	↑ survival rate and proliferation of the cells	↑ EF and FS ↓ area of fibrosis in comparison to the control group	[48]
**overexpression of *YTHDC1* gene**	BMSCs	YTHDC1 inhibits NfƙBiα, and by that regulates apoptosis and reactive oxygen species production	↑ survival rate of the cells	↑ EF and FS ↓ area of fibrosis in comparison to the control group	[49]
**transfecting BMSCs with miRNA-133a**	BMSCs	miRNA-133a inhibits transformation of cardiac fibroblasts into myofibroblasts and reduces collagen deposition	↑ survival rate of the cells	↑ left ventricular ejection fraction (LVEF) ↓ area of fibrosis in comparison to the control group	[51]

↑—increase; ↓—decrease.

**Table 2:** Therapeutic effects of RNAs delivered through exosomes.

Type of Exosomes	Type of RNA	Mechanism of Action	Therapeutic Effects Observed in Treatment Group	References
**ADSCs-exos**	miRNA-205	increasing expression of HIF1a and VEGF, decreasing the level of caspase-3	common: ↑ LVEF, angiogenesis ↓ inflammation, area of fibrosis, apoptosis miRNA-205 ↑ LVFS miRNA-126: ↓ expression of IL-1b, IL-6, TNF-a	[67,80,82,93]
	miRNA-126	enhancing VEGF signalling pathway		
	miRNA-221/222	regulating PUMA/p53/BCL2 pathway and ETS-1/fibronectin/collagen 3 pathway		
	miRNA-671	inactivation of the TGFBR2/Smad2 axis		
**hucMSCs-exos**	miRNA-223	regulation of S100A9 expression, modulation of the P53/S100A9 axis	common: ↑ angiogenesis, LVEF, LVFS, ↓ area of fibrosis, apoptosis, LVID, LVEDD siRNA targeting EGR1: ↑ mitophagy ↓ cytoplasmic cytochrome C levels, number of damaged mitochondria	[71,76,94]
	miRNA-29b	Inhibition of the TGF-β/Smad signalling pathway, reducing expression of MMP-2, MMP-9, collagen type I and III		
	siRNA targeting EGR1	upregulation of Bcl-2, decreasing Bax protein level, increasing the LC3II/LC3I ratio, reducing p62 expression		
**BMSCs-exos**	miRNA-19a/19b	reducing the expression of BIM and *PTEN* genes, reducing expression of collagen type I and III	common: ↑ LVEF ↓ area of fibrosis, inflammation, apoptosis, oxidative stress IncRNA: ↓ LVEDD, left ventricular end-systolic dimension (LVESD), ferroptosis	[16,87,95]
	miRNA-129-5p	inhibiting HMGB1 expression		
	miRNA-29b-3p	inhibiting ADAMTS16 expression		
	lncRNA	upregulating GAS5-mediated UL3/Hippo pathway		
**hiPSCMs-exos**	miRNA-21-5p	increasing expression of Bcl-2, decreasing expression of Bax	common: ↑ LVEF, angiogenesis ↓ area of fibrosis miRNA-21-5p: ↓ apoptosis miRNA-302b-3p and miRNA-373-3p: ↑ cardiac cells proliferation, left ventricular end-diastolic volume (LVEDV), left ventricular end-systolic volume (LVESV)	[96,97]
	miRNA-302b-3p and miRNA-373-3p	regulating HIPPO signalling pathway		

↑—increase; ↓—decrease.

**Table 3:** Minimum reporting items recommended for stem cell and EV studies.

Category	Recommended Minimum Reporting Items	References
**Stem cell source**	Tissue of origin, donor characteristics, autologous or allogeneic origin	[139,140,141]
**Cell culture parameters**	Passage number, culture medium, expansion conditions	[129,140,141,142]
**Cell characterisation**	Surface marker profile, differentiation capacity, viability	[139,140,141]
**Potency assays**	Functional assays demonstrating anti-fibrotic activity (e.g., inhibition of fibroblast activation, reduction in collagen production)	[133,143,144]
**EV isolation method**	Ultracentrifugation, size-exclusion chromatography, precipitation-based methods	[129,141,142,145,146,147]
**EV characterisation**	Particle size distribution, particle concentration, morphology	[129,145,146,147]
**EV markers**	CD63, CD81, CD9, TSG101 and absence of contaminants	[129,141,146,148]
**Dose definition**	Particle number, protein concentration or equivalent dosing unit	[129,145,149,150,151]
**Storage conditions**	Storage temperature, duration, freeze–thaw cycles	[129,142,147]
**Administration protocol**	Route of administration, dosing frequency and timing	[144,145,149,150,152]

**Table 4:** Problems with stem cells and exosome-based therapies.

Type of a Problem	Impact of the Problem on the Therapy:		References
	Stem Cells Therapy	Exosome-Based Therapy	
Immunogenicity/Inflammatory potential	High	Low/variable	[116,154,156]
Bioavailability/Off-target effects	Low	Low	[17,36,159,161,162,163,164,188]
Functional maturity/Predictability	Low	Variable	[165,166,167,168,184,185,186]
Manufacturing complexity/Standardisation	High	High	[65,116,117,118,119,120,187,188]
Lack of clinical evidence	High	Very high	[65,153,162,176,177,189,190]

**Table 5:** Limitations, advantages, and requirements of biological EVs and EVs mimetics.

	Biological EVs	EVs Mimetics
Advantages	Limited or negligible unwanted immunogenicity; effective delivery to target cells [195].	Pure, well-defined systems; lower immunogenicity; reduced production costs; specific biodistribution; cargo stability [196].
Limitations	Roles remain poorly understood in both health and disease; challenges with large-scale production [197]; exosomes derived from aged MSCs exhibit reduced regenerative potential [198].	Lack of an optimised synthesis protocol [197]. Incorporation of multiple proteins is time-consuming and complex [195].
Requirements	Isolation, production, and storage lack standardised protocols; limited availability of reliable assays to evaluate therapeutic efficacy [198].	Quantification and isolation methods remain similar to those used for conventional EVs; new quality standards are required [197].

## Data Availability

The contributions presented in this study are included in the article. Further inquiries can be directed to the corresponding authors.

## Abbreviations

The following abbreviations are used in this manuscript:

ADSCs	Adipose-derived stem cells
ADSCs-exos	ADSC-derived exosomes
BMSCs	Bone marrow stem cells
BMSCs-exos	BMSCs-derived exosomes
CPCs	Cardiac progenitor cells
ECM	Extracellular matrix
EF	Ejection fraction
EVs	Extracellular vesicles
FS	Fractional shortening
hAFMSCs	Human amniotic fluid-derived stem cells
hiPSCMs	hiPSC–derived cardiomyocytes
hiPSCMs-exos	hiPSCM-derived exosomes
hiPSCs	Human-induced pluripotent stem cells
hiPSCs-exos	hiPSC-derived exosomes
hucMSCs	Human umbilical cord MSCs
hucMSCs-exos	hucMSC-derived exosomes
iPSCs	Induced pluripotent stem cells
iPSCs-exos	iPSC-derived exosomes
LVEDD	Left ventricular end-diastolic dimensions
LVEDV	Left ventricular end-diastolic volume
LVESD	Left ventricular end-systolic dimension
LVESV	Left ventricular end-systolic volume
LVEF	Left ventricular ejection fraction
LVFS	Left ventricular fractional shortening
LVID	Left ventricular inner diameter
MI	Myocardial infarction
MSCs	Mesenchymal stem cells
